# Very rapid insight generation to support UK health and care systems: An AHSN approach

**DOI:** 10.3389/fsoc.2023.993342

**Published:** 2023-03-28

**Authors:** Jackie Chandler, Philippa Darnton, Andrew Sibley

**Affiliations:** Wessex Academic Health Science Network, Southampton, United Kingdom

**Keywords:** rapid evaluation, COVID-19, rapid insight, AHSN, National Health Service (NHS)

## Abstract

**Introduction:**

COVID-19 challenges are well documented. Academic Health Science Networks (AHSNs) are a key partner to NHS and care organizations. In response to managing COVID-19 challenges, Wessex AHSN offered rapid insight generation and rapid evaluation to local NHS and care systems to capture learning during this period. This novel “Rapid Insight” approach involved one-off online deliberative events with stakeholders to generate insights linked to specific, priority areas of interest, followed by rapid analysis and dissemination of the findings.

**Context:**

Key objectives were to enable system leaders to build their adaptive leadership capability and learn from the experience of COVID-19 to inform recovery planning and system support. Rapid Insight (RI) gathered together health and care professionals into a tightly managed, virtual forum to share system intelligence.

**Approach:**

Focused questions asked about the systems' response to the pandemic, what changes to continue and sustain, or discontinue. Participants responded simultaneously to each question using the virtual chat function. Immediate thematic analysis of the chat conducted in 48–72 h by paired analysts for each question strengthened analytical integrity. Mind maps, the key output, provided easily assimilated information and showed linkages between themes. Telephone or virtual interviews of key informants (health and care professionals and patients) and routinely collected data were synthesized into short reports alongside several RI events. However, insufficient time limited the opportunities to engage diverse participants (e.g., mental health users). Data from RI can scope the problem and immediate system needs, to stimulate questions for future evaluative work.

**Impact:**

RI facilitated a shared endeavor to discover “clues in the system” by including diverse opinions and experience across NHS and care organizations. Although these rapid virtual events saved on travel time, digital exclusion might constrain participation for some stakeholders which needs other ways to ensure inclusion. Successful rapid engagement required Wessex AHSN's existing system relationships to champion RI and facilitate participant recruitment. RI events “opened the door” to conversations between up to 150 multi-professional clinicians to share their collective response to COVID-19. This paper focuses on the RI approach with a case example and its further development.

## 1. Introduction: NHS and care system leaders' need for rapid learning to respond to COVID-19

COVID-19 created unprecedented levels of disruption particularly to the access and delivery of health and care. The NHS in the UK needed real time information from multiple sources in manageable formats. Wessex Academic Health Science Network (AHSN) responded to local health and care system leaders' needs to adapt quickly to the pace of change demanded by the COVID-19 pandemic from March 2020 and designed a virtual rapid information feedback cycle. Rapid Insight (RI) brings together members of the NHS workforce and staff from other sectors (e.g., adult social care, care homes, and voluntary sector organizations) from across their local systems to provide an opportunity to reflect and share lessons and knowledge about a common focus or practice change whilst it unfolds. The success of rapid evaluations requires well established relationships, which enables accessing the right people and collecting the right data.^1^ Positioned to do this, AHSNs sit at the nexus of multiple agencies and build strong relationships with their local health and care systems.

NHS health and care systems needed to manage the impact on patients and NHS services as a result of the pandemic due to treatment backlogs, delays in diagnosis, and workforce challenges (Reed et al., 2022). The pandemic crisis presented an opportunity to better understand emergent new ideas for ways of working, and the potential for ongoing change to address system weaknesses exposed by the pandemic to a post-crisis state (Taylor, 2020). Wessex AHSN's initial focus was to enable system leaders to build their adaptive leadership capability (Heifetz, 1994; Liles and Darnton, 2020) and learn from the experience of COVID-19 to inform pandemic recovery planning. This also required an approach that brought leaders across health and care together to promote collaboration as an effective tool to facilitate rapid and innovative decision making (Horwood et al., 2022).

Rapid qualitative and mixed method evaluation approaches, in particular, have a history of development with different techniques emerging (Scrimshaw and Hurtado, 1988; Beebe, 2001; Vindrola-Padros and Johnson, 2020; Vindrola-Padros et al., 2021). Rapid evaluations are characterised as participatory, team-based, iterative and lasting from a few weeks to a few months (McNall and Foster-Fishman, 2007). They typically involve shortening of timescales and methods (Schünemann and Moja, 2015; Vindrola-Padros et al., 2021). The COVID-19 pandemic has particularly engaged rapid evaluation approaches to provide timely information feedback to the healthcare system (Vindrola-Padros et al., 2020; Ramsay et al., 2022; Singh et al., 2022).

**Figure 1:**
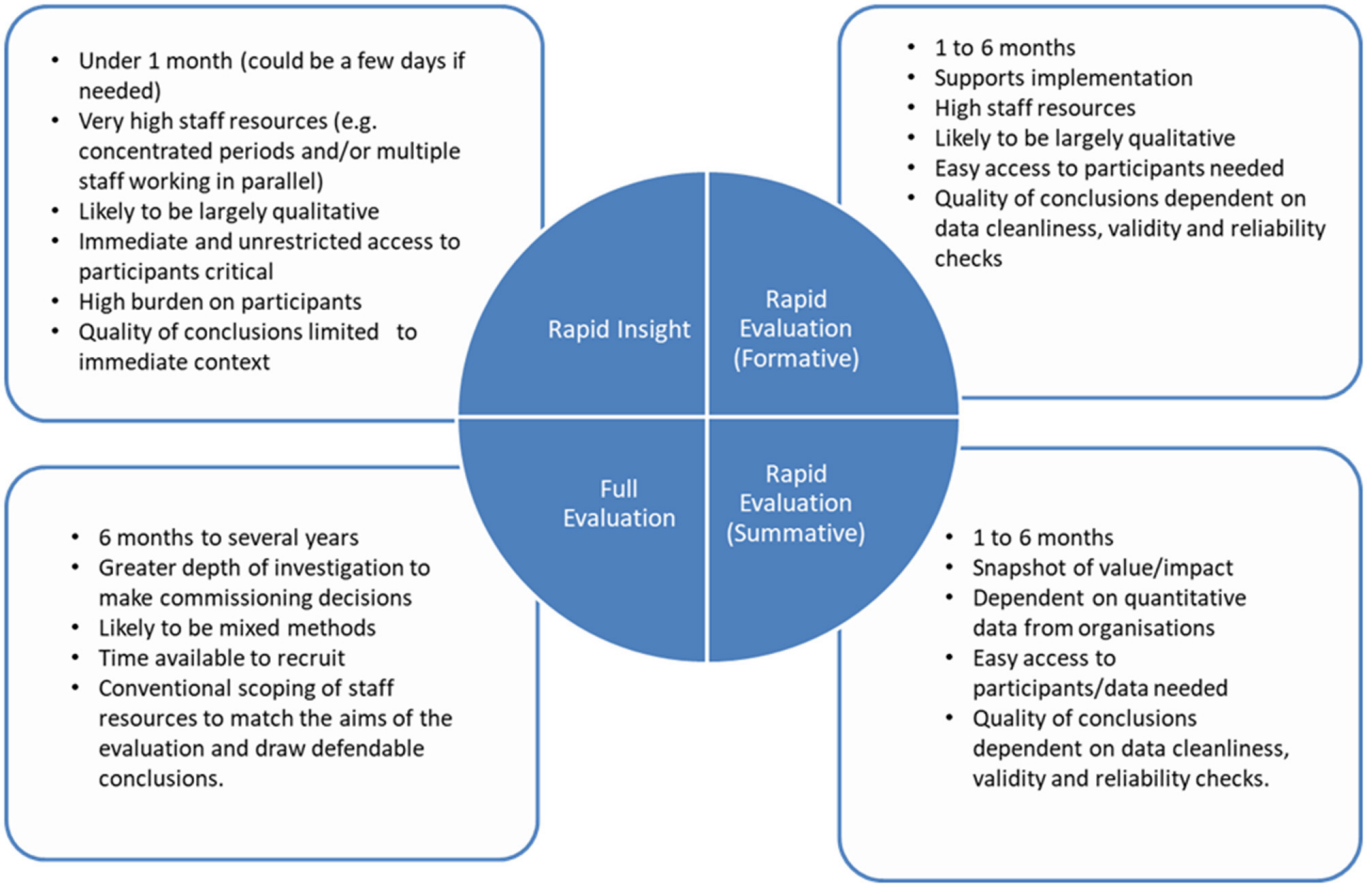
A basic positioning of Rapid Insight within the evaluation landscape.

The RI approach is experimental but popular with local health and care organizations covered by Wessex AHSN and has generated interest from the wider AHSN Network in England. A basic comparison between Rapid Insight and other forms of evaluation is presented in Figure 1, to support the positioning of RI in the evaluation landscape. RI is especially differentiated by its speed and shortening of methods including approaches to data collection and analysis and sits within the continuum of other rapid qualitative approaches (Vindrola-Padros et al., 2021). The first RI event occurred in June 2020. This article will discuss its development, the RI process, practical implications for learning and potential for future development.

## 2. Context: Development of rapid insight for health and care system stakeholders

The important role of AHSNs in the UK context was a key factor in the development of RI. Wessex AHSN was established in 2014 and is part of a connected national network of fifteen similar networks working across England. The Academic Health Science Network (AHSN Network) is commissioned by NHS England and the UK Government's Office for Life Sciences to foster the uptake and spread of innovation to improve health and generate economic growth. AHSNs achieve this by connecting the NHS, academic organizations, local authorities, voluntary and other non-profit agencies, and industry (commercial organizations) to create the right conditions to adopt innovation. They have first-hand experience of supporting the implementation of innovations in response to the pandemic in acute, community and primary care sectors (e.g., telehealth and COVID-19 virtual wards).

Wessex AHSN works within a region in southern England covering the counties of Hampshire, the Isle of Wight, Dorset, and south Wiltshire. It has a strong record of working both locally and nationally to support the adoption and spread of innovation, including an established Insight function, and drew upon a range of skills to design the RI approach. This includes specialist knowledge about innovation in the NHS, identification of innovations, and their adoption and spread in NHS organizations (Wessex AHSN, 2019; Sibley et al., 2021). The AHSN's Insight function is provided by evaluation practitioners with expertise in conducting process and impact evaluation, real world evaluations and implementation science.

NHS and care leaders needed accurate, detailed, and actionable feedback within a short timeframe to match the speed of change and need for adaptation to enable accelerated decision making and large-scale implementation of change. Leaders needed the AHSN to produce information in a highly structured, summarised and easily assimilated format. This allowed them to use the findings in their management of health care challenges which required system level decision making.^2^

The team identified the importance of Heifetz's work on responding to adaptive challenges and the nature of leading when there are no easy answers (Heifetz, 1994). This became the frame of reference which guided the development of RI, as follows:

1. It was clear that the level of disruption caused by the COVID-19 pandemic would mean the health and care workforce organizations in Wessex, England, UK would face very significant adaptive challenges over the coming years. So those that build their adaptive capacity now, will be best placed for this challenge.
2. Adaptive challenges require adaptive responses to reduce the gap between the values people stand for and the new reality they face. Testing and understanding the reality of the challenge means involving people from multiple vantage points and not just through normal lines of authority.
3. It was clear early on, that health and care organizations would not be able to get back to what was “normal” before the pandemic. The RI approach therefore needed to help these organizations to understand their adaptive challenge and response in ways that would help them manage this long period of unprecedented disruption to adapt to and understand their “new normal”. (Liles and Darnton, 2020)

Challenges for NHS and care leaders, at the time, included early discharge to free up beds, the move towards remote consultations and delivering vaccines. In response, examples of RI events that provided insights were a regional vaccination programme, regional implementation of COVID-19 virtual wards, staff wellbeing, digital self-care solutions in primary care, and these informed the development of digital strategies and innovation priorities. As the RI programme of work progressed, weekly oversight meetings took place to reflect on each engagement, and to rapidly appraise what worked and what could be improved. At the height of the pandemic, there were monthly RI events. Insight's evaluation methodologists reflected on the RI approach and other rapid evaluation methods used by the team during the COVID-19 response, as they evolved to understand RI's place in the spectrum of evaluation approaches.

In the 2 years since June 2020, the AHSN has developed a standardised process for managing and running these virtual events. This enabled the participation of over 700 people from a range of health and care organizations and the public, and up to 150 in a single event. Each event had senior sponsorship from the partnering organisation(s).

## 3. Approach: Principles and procedure of rapid insight

This section describes seven equally important principles which guide the operationalisation of the RI approach (see Box 1).

Box 1 **Principles of rapid insight to guide event planning.**

**Seven principles for successful RI events**
**1. Allocate appropriate resources**
• RI events require adequate numbers of evaluators, many with both evaluation and local NHS system context knowledge and experience.
• Planning, preparation for the event and intense analysis following it require time allocated in diaries.
**2. Mentally prepare to work at pace and as much depth as possible**
• RI events are intense and require evaluators to adopt a rapid mindset.
• RI event analysis requires evaluators to adopt pragmatic approaches that maximise depth with speed to provide high quality outputs.
**3. Seek and sustain enthusiastic and timely local engagement**
• Success of RI events requires both the system leads participating and the evaluators to co-operate effectively. In particular, accessing and engaging participants.
• Clear expectations of engagement and commitment should be outlined following an agreement to run an RI event.
**4. Seek and sustain a tight focus of investigation**
• The problem or topic of interest needs to be tightly focused.
• Question formulation is critical to maintain focus and manage system lead expectations.
• RI event findings are context specific.
**5. Plan and manage the technical requirements of remote data collection**
• Successful RI events require appropriate virtual platforms that can manage large numbers of participants (e.g., 100 or more).
• Administrative and technological support is required to manage the RI event.
• Participants require clear instructions on the process and expectations to produce information *via* the chat function.
**6. Work in teams to fast-track data collection and analysis simultaneously**
• Simultaneous working is key to running an RI event and any other data collection activities (e.g., patient interviews) to optimise the speed of RI planning, data collection, analysis, and writing. Organising teams to complement each other's skill sets and have a proven ability to work quickly together will ensure the Rapid Insight deadline is met.
**7. Use triangulation to increase validity/reliability/richness of findings**
• RI events can be used as standalone events; however, supplementary data collection activities can enhance the validity of the RI event findings.
• Multiple evaluators provide a verification of the data.

**Principle 1: Allocate appropriate resources**

Rapid evaluations, typically, require careful consideration of resources because timeliness of findings is crucial. The RI approach, specifically, is quicker and seeks feedback within 48–72 h to 1 week to make findings timely for any urgent decision-making processes. Therefore, more speed means more resources to operate fully the event, complete the analysis, discuss the findings, and write up the report. An event with 100 participants, for example (see case example), could involve at least 11 members of staff: one senior member of staff as the main host to guide the whole event; two members to manage the technical planning, develop an event running order and plan, and manage queries on the day across multiple online groups; and two analysts for each major question the event is addressing (typically around four questions). In addition, each event has appointed “observers” who monitor the chat thread providing some initial verbal feedback to the participants. The analysts may be very experienced AHSN staff or have a clinical background or evaluation experience. They would be briefed in advance of the event and paired with a more experienced RI analyst to conduct the rapid analysis work in the days following the event. Crucially, the role of the chair is an important element to the RI event, requiring considerable effort, energy, and effective time-management to ensure the concert of activity is conducted in harmony.

**Principle 2: Mentally prepare to work at pace and depth**

As RI events are fast and intense, staff need to prepare and commit the necessary time allocated to event preparations, the event and subsequent analysis. The rapid flow of the RI procedure is important to ensure the quality of the findings. These RI events are tightly managed and require a dedicated focus, for up to 48 h, to achieve as thorough a qualitative analysis as possible.

**Principle 3: Seek and sustain enthusiastic and timely local engagement**

The nature of RI events are symbiotic. The quality of the outputs depends heavily on the engagement of stakeholders facilitated through careful support and preparation before the event. This ensures they understand expectations of how the event will run. Facilitators need to inspire participants with enthusiasm and energy to encourage participation. Fortunately, the focus of the event is usually about an important and timely issue leading to highly relevant insights.

**Principle 4: Seek and sustain a tight focus of investigation**

Ensuring a tight focus of investigation is a critical component due to the form of the event. All those using the approach should avoid addressing overly complex questions. This will help to generate insights that are actionable, e.g., by informing decisions about future ways of working—what to adapt, improve or discontinue for the benefits of patients and staff. A focus also helps ensure the timely production of outputs, as analysts have a boundary around the event and reasonable expectations for the 48 h post-event period. Thus, as in all forms of evaluation design, question formulation is an important element that takes effort and consideration involving both those commissioning and facilitating the event.

**Principle 5: Plan and manage the technical requirements of remote data collection**

The short timeframe for the RI event is deliberate and a critical factor. RI events usually run between 1 and 2 h depending on the number of questions, and number of participants anticipated. A range of innovative technical solutions need to ensure fast data collection and an easily manageable data set after the event. Due to time constraints and the fast pace of question, reflection and response, detailed event planning will assist the organisers and participants. This also requires a clear running order with run-throughs with all participating members of staff. Staff facilitating the RI event need a task list to ensure all staff involved in the event maintain the timeliness of outputs. These RI event processes have been standardised to ensure a common approach and consistency of quality outputs.

**Principle 6: Work in teams to fast-track data collection and analysis simultaneously**

Pairs of analysts will jointly present and manage activity on a question and manage the outputs rapidly after the event. By working in pairs, staff throughout the process will develop familiarity and continuity between the question responses and their subsequent analysis. Importantly, all pairs of analysts must work simultaneously and independently the day after the event. The nature of the qualitative coding and theming is semantic, to identify the explicit and surface meanings of the data. To support the speed of analysis and reporting, analysts schedule regular review calls throughout the day. To expedite analysis, all comments are reduced to short paraphrased statements. These are subsequently collated to develop first and second order themes which may include categories rather than themes, where appropriate. In addition, identifying different stakeholder perspectives is also done within the mind maps. Further discussion on analysis and participation is discussed in Section 4.

**Principle 7: Use triangulation to increase validity, reliability and richness of findings**

At the end of the rapid analysis period, analysts should meet as a group to develop and agree the final mind map output. This presents an opportunity to increase the qualitative trustworthiness of the findings, primarily using techniques such as peer debriefing and team consensus on themes (Nowell et al., 2017). In some situations, checking findings with the commissioner of the RI event should also be done. It is noted that triangulation should be used to ensure findings are as rich, valid, and reliable as possible, and so there are benefits to the collection of additional qualitative data that provides more discursive findings than the RI event output.

**Figure 2:**
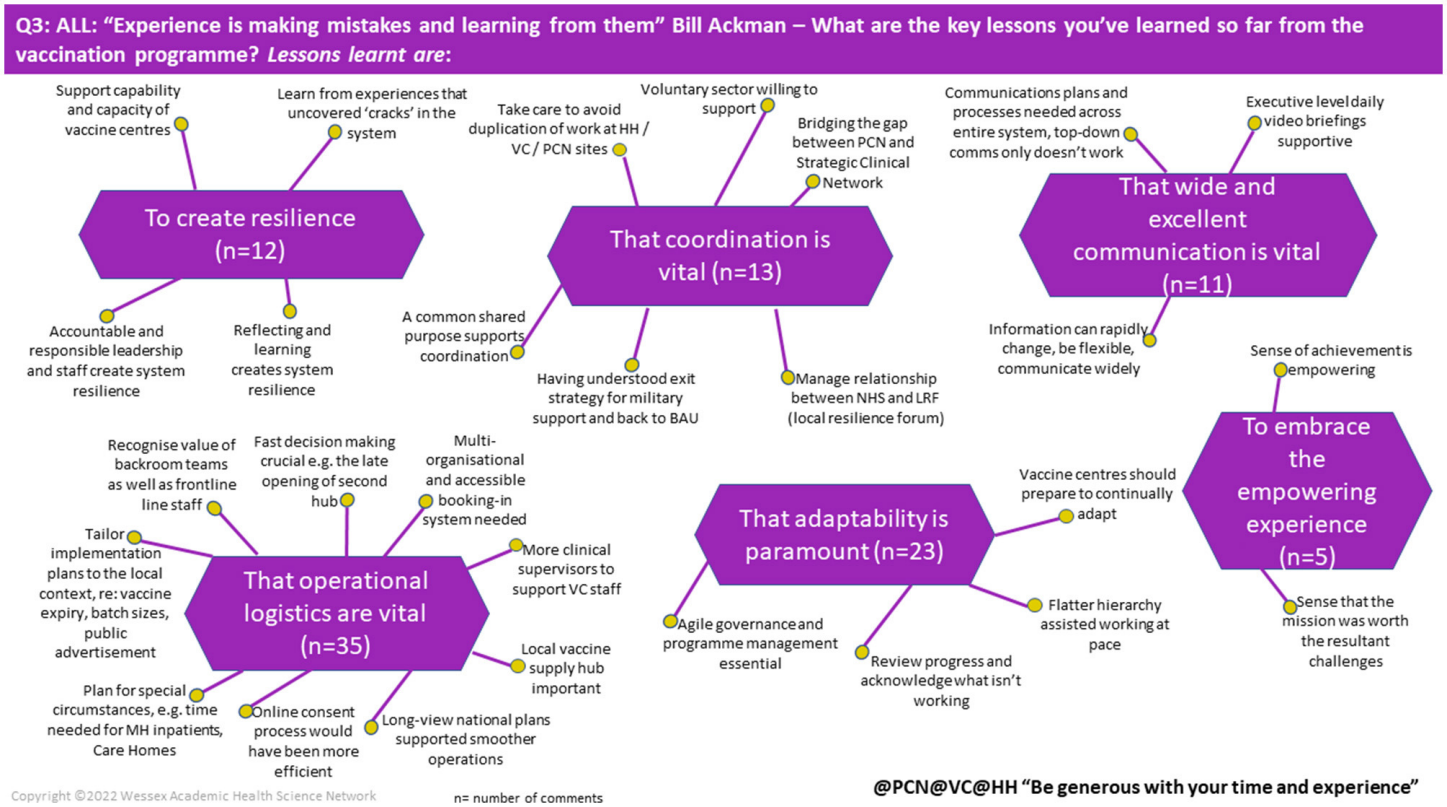
Example of mind map. Extracted and modified from RI Report: NHS England—South west region rapid insight and learning from the COVID-19 mass vaccination programme—NHS England—South west region rapid insight and learning from the COVID-19 mass vaccination programme, Wessex AHSN, April 2021.

Using all these principles can ensure both rapid and in-depth insights on the issue investigated are summarised effectively into a mind map of findings (see Figure 2).

Importantly, RI needs tailoring to the context under study, e.g., the problem, the questions, the number of participants involved, the extent of technical online support, and the composition of analysts to manage the outputs of the RI event. The RI case example describes an event to glean learning from the COVID-19 mass vaccination programme in the NHS England—South West region that took place in April 2021.

Finally, all invited participants are provided with pre-event information about the topic and purpose of their participation. Rules of confidentiality and consent are displayed at the beginning of the group. Data from the event is anonymised. Any risk of identification is checked, and additional consent requested to proceed with publication, if required.

### 3.1. Rapid insight case example—NHS England-South West Region rapid insight and learning from the COVID-19 mass vaccination programme

**When:** April 2021.

**Who:** 102 participants including front line staff, operational managers and service and strategic leaders from the mass vaccination programme across NHS England—South West Region, UK.

**Questions:**

1. Thinking about when you were asked to set up the vaccination programme, from your perspective, “**What went well?**”
2. Thinking about when you were asked to set up the COVID-19 vaccination programme, from your perspective what could have been “**Even better if?”**
3. “Experience is making mistakes and learning from them.” Bill Ackman (2020)— “**What are the key lessons you have learned so far from the vaccination programme**?”
4. “**What factors need to be in place to create a sustainable vaccine service for the future**?”

**Method:** Purposively invited participants from all relevant agencies involved in vaccine administration attended a single virtual event on a remote platform. A large team of facilitators, administrators and analysts supported the RI event. Participants were guided through the key steps to enable them to respond to the questions and comment in the remote platform chat. Questions were sequentially presented and two analysts per question reflected initial key points during the event to the participants. Careful instructions and monitoring throughout the event guided participants to think and reflect and then respond in the chat. Participants were asked to tag their comment with six agency identifiers, e.g., primary care networks (@PCN), vaccine centres (@VC), community pharmacy (@CP). Chat feed was downloaded into an Excel document by question. Pairs of analysts conducted a simplified thematic analysis of this feed. First order and second order themes were put into a mind map structure for presentation of findings to those who commissioned the RI event. Responses were presented by tag for questions 1 and 2, all responders for question 3 and question 4 by responders and strategic level across the health system.

**Findings:** Analysis of the chat feed was conducted over 2 days immediately post the RI event. Table 1 provides first order themes and the number of comments supporting the theme. Figure 2 illustrates 1 of 11 mind maps produced for this RI event.

**Table 1:** Questions and first order themes for the rapid insight case example—NHS England—South west region rapid insight and learning from the COVID-19 mass vaccination programme.

**Question**	**First order themes (combined across all agencies)**	**No. comments**
What went well?	A united supported workforce	30
	Strong leadership and permission to act	28
	System collaboration to deliver at pace	58
	Shared purpose, vision, and culture	31
	Operational logistics and technology	13
	Shared learning and communication	9
What could have been better if?	Better communication and liaison	38
	Improved system culture	31
	Launch of vaccination model	23
	Development of interoperable IT and booking systems	19
	Ongoing delivery of vaccination model	25
What are the key lessons you have learnt so far from the vaccination programme?	Create resilience	12
	Coordination is vital	13
	Wide and excellent communication is vital	11
	Embrace the empowering experience	5
	Adaptability is paramount	23
	Operational logistics are vital	35
What factors need to be in place to create a sustainable vaccine service for the future?	Supported and flexible workforce	28
	Development of communication strategies	10
	Creating future sustainability together	25
	System planning	34
	Vaccine logistics	34
	Consider the needs of hospital hubs, vaccine centres and primary care	85

**Limitations:** Findings are limited to the specific NHS England—South West region, participants that attended and the specific timepoint of the RI event and should be corroborated with other data to inform decisions.

### 3.2. The limitations of the rapid insight approach

RI events are time sensitive, and findings present a snapshot based on the views of those present. Differences in time, setting and participants is likely to lead to different findings. No formal evaluation or impact assessment has yet determined the added value of the RI approach. Informally, feedback from commissioners of the RI events suggests that they continue to have currency for local NHS and care systems. Some issues and reflections are described below.

#### 3.2.1. Methodological limitations

The depth to which RI events are able to capture views and insights is limited and the benefits of RI information can be further strengthened if other data collection can inform, triangulate, and surface any counter perspectives. RI events present a snapshot of reflective views based on the perspectives and recall of the participants that contribute. The simplified thematic analysis approach is discussed later here and does not intend to reflect more intense approaches such as Braun and Clarke (2006, 2019). Findings are exploratory and not conclusive. They are context specific and bounded by time and place.

#### 3.2.2. Technological limitations

Overall, management of the remote platform evolved procedures that were reproducible, and responsibility for the technical smooth running of these events was assigned to one individual as a key task. Participants would be given clear instructions on the process of the RI event. However, various issues might occur in some sectors such as care homes due to lack of IT facilities and therefore, remote access to all relevant stakeholders for an event cannot be assumed and other methods of participation should be considered.

#### 3.2.3. Human online interaction limitations

Two key limiting non-technological aspects were non-responders, those that attend but make no contribution, and second the lack of interaction between responders. Although, a key benefit of RI events was facilitating less heard voices which allowed greater diversity of experience. An informal count across several RI events attended by the lead author indicated the responder rate was between 30 and 50%, irrespective of whether there were 50 or 100 participants present. Individual participants are able to dominate in this type of event as with any other by repeatedly entering into the chat function. In addition, as with all data collection activity involving human participants, people can misunderstand instructions. One mitigation, added to the process, was to follow up absent participants or those that do not contribute and offer the opportunity to complete a post event form with the questions to return by email. The one-way communication denied the option to discuss and elaborate because participants do not interact as expected in a focus group (Finch et al., 2003).

## 4. Impact: Early indications of the rapid insight approach

These events are beneficial as they bring key stakeholders together at system level, thereby providing a means to support wider stakeholder contributions as transformational changes take place in the NHS with Integrated Care Boards and Integrated Care Systems. These systems seek to integrate health and care services and build relationships and joint strategies between local authorities and NHS commissioners in England. The RI approach could produce a form of policy evidence when reviewing strategic needs.

A short impact survey was sent to key stakeholders who had attended the early events to understand their experiences and the impact of the RI approach, and to inform the development of the approach. This survey was sent to the key stakeholders for seven events (i.e., those who commissioned the work), twice during 2020/early 2021. Unfortunately, only two responses were received no doubt, in part, due to the continuing pressures at the time. The first 12 months of the programme was also reviewed for The Health Foundation (joint funders of the programme) which additionally reported the views of AHSN staff who had been involved in delivering the events (Box 2).^3^

Box 2 **Reflections from senior health leaders and the Wessex AHSN team.**

**Reflections from senior health leaders captured through an impact survey:**

“*(The programme) brought our system together with a shared appreciation of what has happened. Provided some qualitative data on what has worked and why. Enabled people to reflect on what they want to keep going forward.”* (a partner)

“*The rapid insight work enabled us to understand a really diverse set of views and brought us together as a system around some very concrete shared experiences. It has accelerated our culture of learning together as a system.”* (a partner)

“*It provided a forum for honest reflection and discussion and allowed us to come up with some common areas that we must take forward. The easy and relaxed style broke through the usual hierarchy we encounter, and everyone was able to have a voice and participate which allowed everyone to contribute equally. It was a great session and has given us some clear areas of work to focus on together, as well as a new way of working to develop insights. Thanks to the team for leading us through this.”* (a partner)

**Reflections from the Wessex AHSN team requested by the programme lead:**

“*The programme has been hugely rewarding to be part of, both in terms of supporting our system partners with learning from the COVID and what to take forward into the future, but also working with the Health Foundation colleagues and the internal AHSN team. I have also thoroughly enjoyed developing new skills and broadening my knowledge about the literature around adaptive leadership.”* (Deputy Chief Executive and Director of Innovation Adoption, Wessex AHSN)

“*Being involved in developing a rapid insight approach has productively challenged my existing understanding of evaluation and generated a new complementary method in the toolkit of evaluation techniques.”* (Evaluation Programme Manager, Wessex AHSN)

“*The Rapid Insight offer helped more of* o*ur system partners see us as valued colleagues, who can practically support transformation. It has made it easier for us to bring evaluation thinking into their planning processes*.” (Director of Insight, Wessex AHSN)

“*I really enjoyed working with our partners in this way. To gain so much information within an hour, providing such rich insight from so many people at such a busy time, was incredible. It also felt like a really fun way to engage. The events themselves offered some time to reflect, in amongst the chaos of the first wave. I'm really looking forward to taking forward this approach in my future work.” (*Associate Director, Wessex AHSN)

“*It was inspiring to collaborate with our partners in this way and to see immediate impacts from the work. I developed new knowledge about adaptive leadership, virtual technologies and facilitating virtual events, and about different ways of presenting information (e.g., mind maps).”* (Associate Director, Wessex AHSN)

“*Working in a new way to support colleagues across our systems was really rewarding—we would not have been able to coordinate diaries across 40–50 people who are diversely spread across our geography without this new approach. I not only learnt a lot personally in terms of successful engagement approaches, but this opened up possibilities for using similar approaches on other programmes I deliver—continue to use touches of the Rapid Insight method in our national delivery programme 9 months later.”* (Associate Director, Wessex AHSN)

The AHSN has not yet undertaken a formal evaluation. Future credibility and validity of the approach and its findings would benefit from understanding the benefits and challenges of RI in the health and care context and benefits to NHS and care leaders who need adaptive approaches to manage complex systems in a complex world (Uhl-Bien, 2021).

### 4.1. RI's position and potential impact in the rapid evaluation field

RI events were a case of “necessity is the mother of invention”. It was a strategy to engage efficiently NHS and care leaders during the COVID-19 pandemic to enable reflective practise in a fast-moving situation for those with responsibility for making decisions. RI is demand led by the NHS, and therefore typically addresses more pragmatic questions than research questions (that are broader).

In addition to the basic comparison of RI in the evaluation landscape in Figure 1, this section further explores its position and value within the range of evaluation methods. RI events are based on good evaluative practise and have a framework that includes question formulation, participant eligibility and selection, and rigour of data analysis with two analysts cross checking data and reaching agreement on themes. Nevertheless, although a standalone technique, findings need substantiating alongside other data collection activities. Considerations as to where RI events might fit into more typical qualitative data collection methods suggest it is neither an interview, a focus group nor an observation. Nevertheless, the approach is structured, participants are purposively selected, and evaluation type questions asked. Vindrola-Padros et al. (2021) identify multiple rapid evaluation approaches in their systematic review. However, the review found data were collected using typical quantitative or qualitative methods over various timeframes, the shortest duration 3 months. RI events feedback findings to those who commissioned the event and through the AHSN website within 48 h to a week and so are more rapid than other rapid approaches (Vindrola-Padros et al., 2021). RI events might fit within quality improvement techniques because the approach answers specific questions, provides opportunity for iterative feedback loops, focuses on priorities, captures change, and shares intelligence within a system.^4^

RI events bring together in one space all principal and relevant stakeholders from across a specific healthcare system to address a set of focused questions. Availability of these stakeholders is limited, and they are often time poor. Nevertheless, as the case example illustrates despite pressures at the time on the system, this RI event had particularly good uptake. Successful events have engaged the right people and provided the opportunity to draw together the opinions and experiences of influential decision makers from the local health care systems. The approach is spontaneous, questions are not provided beforehand and although participants can see each other's responses they do not normally engage with each other on the questions and each comment represents a personal reflection. However, the influence of participants on each other cannot be eliminated.

### 4.2. RI and evaluation timeframes

“Rapid” in evaluation and research (primary or secondary) typically means to shorten evaluation timescales (see Figure 1), which requires more human resources and truncation of methods (Schünemann and Moja, 2015; Vindrola-Padros et al., 2021). RI events adopt both. More human resources are provided from administrative and technological input to qualitative analysts. Also, as a rapid approach it truncates other typical qualitative methods such as focus groups and adopts a simplified approach to thematic analysis. Therefore, the approach is heavily reliant on human resources to support it. However, high person hours are only maintained for a short period of time.

### 4.3. RI and thematic analysis

Rapid thematic analysis as described requires people to synthesise rapidly the data into themes and produce thematic mind maps. A methodological study (Taylor et al., 2018) compared thematic and rapid analysis techniques on the same qualitative material by different research teams. Outcome measures were time taken to complete analysis in person hours; whether analysis findings and recommendations matched, partially matched, or did not match across the two teams in the study. Study authors report rapid thematic analysis delivered valid findings that overlapped with the traditional thematic analysis and showed that rapid thematic analysis enabled considerable time savings in data management by up to 2 weeks. However, time for interpretation and finessing findings for reporting took longer in the rapid analysis approach. In this study, a key limitation was differences in researcher approach to analysis. The traditional thematic analysis was conducted by one researcher, therefore less opportunity for discussion and reflection. Rapid analysis had more researchers involved who shared an office space providing opportunities for regular reflection (Taylor et al., 2018). Nevedal et al. (2021) in a qualitative analysis comparative study compared rapid and traditional qualitative analytical approaches and demonstrated transcription savings and reduction in analyst hours, however, data interpretation was no different across approaches. Both these studies indicate that qualitative discussion and agreement across analysts is not so easily reduced.

In contrast, the thematic analysis process in RI events involves a team of people working intensively together in pairs to produce a final product over 48 h with edits for a final report taking up to a week. Analyst pairings, therefore, permit discussion and agreement on themes within the compressed timeframe.

### 4.4. RI and reporting findings

Balanced reporting whether research, service evaluation or a RI event is important for those making judgements based on that information.^5^ Reporting of RI events may need further consideration in this respect. Participants are purposively selected to represent the context and “the problem”. Reasons for participating and subsequently not contributing can only be speculation at this point. Currently, the mind maps report the number of comments related to the development of a theme (Figure 2). Reporting of RI events would benefit from separating those in attendance, those that participate (provide comments in the chat) and the number of comments attributable to any one individual participant for each question. One participant can provide multiple comments and reporting needs to reflect the representativeness of participants present to improve methodological quality. In addition, there is little space to provide examples of content such as direct quotes from the chat. Therefore, careful selection is required.

### 4.5. Reflections on the RI approach

Over twenty events have now occurred with more planned and therefore the RI approach has shown utility to local health and care systems. The current approach has become established and future development options are being considered and explored. First, RI might form into a consensus building technique (Briggs et al., 2005) and the approach used to develop recommendations for the local system amongst stakeholders. Second, it recently provided an additional data collection device within a standard evaluation by gathering a broad range of stakeholders to inform this evaluation's ongoing data collection, formatively. Third, it will be used to engage evaluation stakeholders towards the end of an evaluation for participants (key stakeholders in the evaluation) to consider and review summative evaluation findings. Thus, RI is a technique for gathering information and perspectives from a wide range of stakeholders to address a focused set of open questions, which may be undertaken more than once with the same group of stakeholders to understand perspectives over time.

While standalone events were a pragmatic approach during the COVID-19 pandemic, health and care systems would benefit from revisiting the findings of previous events and discerning what has changed, what benefits or impacts previous reflections led towards and an opportunity to update those findings. This could develop and reflect a multi-cycle approach (McNall and Foster-Fishman, 2007), which along with other data collection could enhance the benefits of the RI approach.

Potential considerations for the future development of the RI approach involve (1) a synthesis of learning from previous RI events with findings from the evidence base on rapid evaluation, (2) development of research questions to investigate further the deployment and impact of RI method in different contexts, and (3) collaboration with like-minded NHS professionals, academic colleagues, and teams involved in research on rapid evaluation.

There are clearly benefits to this approach and an appetite for faster insight generation by busy senior leaders of health and care services. However, there are also important limitations to acknowledge and knowing how and when to deploy this approach is important. No formal evaluation of the RI events has yet been undertaken and this is an important next step to understand their popularity, uptake, and impact on decision-making and patient care.

## Data availability statement

The original contributions presented in the study are included in the article/supplementary material, further inquiries can be directed to the corresponding author.

## Author contributions

PD and JC drafted and submitted the abstract. JC led on the manuscript draft and content structure. JC, PD, and AS contributed to the manuscript. All authors reviewed and responded to peer review comments and agreed submitted manuscript.

## Footnotes

^1^ https://www.ahsnnetwork.com/rapidevaluation

^2^https://wessexahsn.org.uk/videos/show/379 - vlog from North and Mid Hampshire describing the benefits to them of RI.

^3^Q–Rapid insight programme final report, February 2021, unpublished.

^4^ https://www.england.nhs.uk/wp-content/uploads/2022/01/qsir-pdsa-cycles-model-for-improvement.pdf

^5^ https://www.equator-network.org/about-us/what-is-a-reporting-guideline/
